# Supplementary material for: Linc00673-V3 positively regulates autophagy by promoting Smad3-mediated LC3B transcription in NSCLC
Source: Life Sci Alliance. 2024 Mar 25;7(6):e202302408. doi: 10.26508/lsa.202302408 (PMC10963591; doi:10.26508/lsa.202302408)

**Figure 3D source data file**

**A549 original blots:**

p62


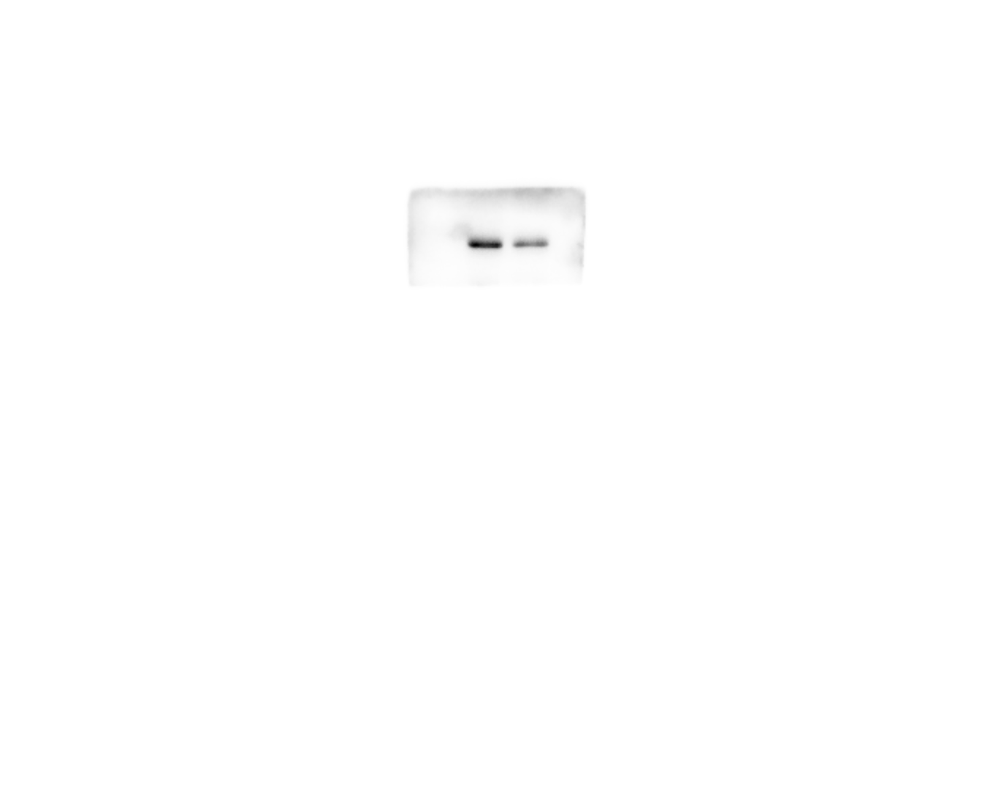


LC3B

nc 0.25 0.5 0.75ug


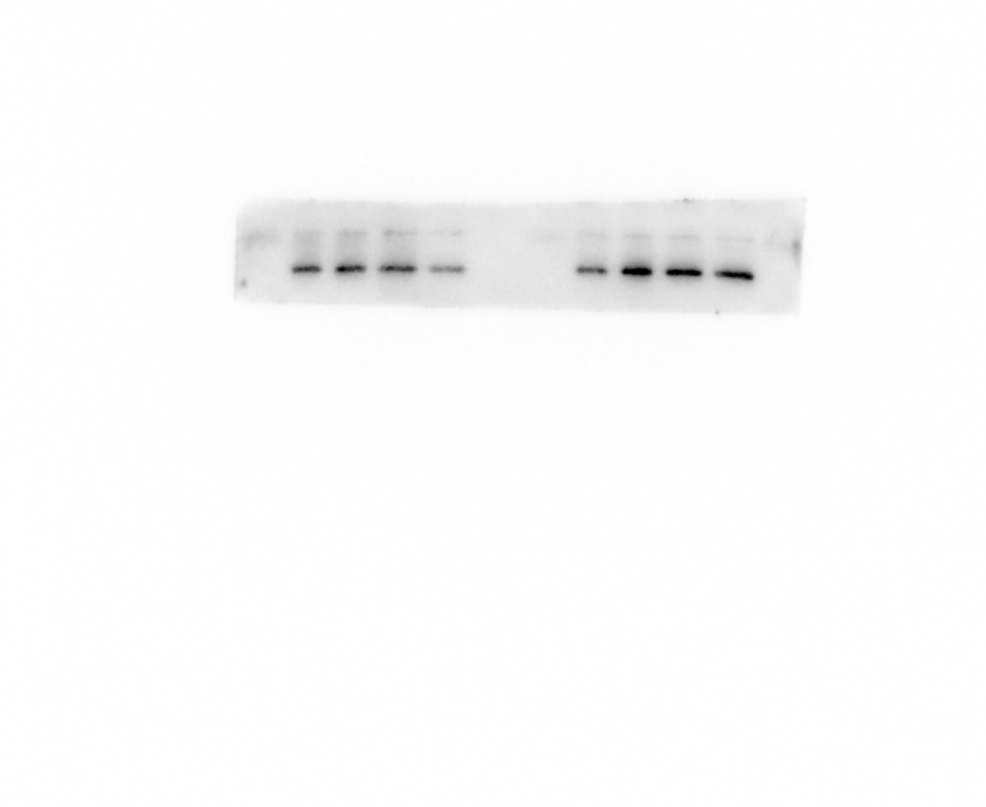


In assessing the impact of overexpressing Linc00673-V3 on the protein level of LC3B in cells, we first conducted a pilot experiment with a gradient of plasmid transfection concentrations. Specifically, we transfected cells with 0.25 μg, 0.5 μg, and 0.75 μg of Linc00673-V3 overexpression plasmids. The results revealed that transfecting with 0.25 μg of the overexpression plasmid was sufficient to stimulate an increase in intracellular LC3B protein levels. Consequently, for subsequent experiments, we chose 0.25 μg as the plasmid transfection concentration. To maintain consistency with the subsequent experimental results, we have selected the first two lanes for presentation in Fig. 3D of the manuscript.

GAPDH


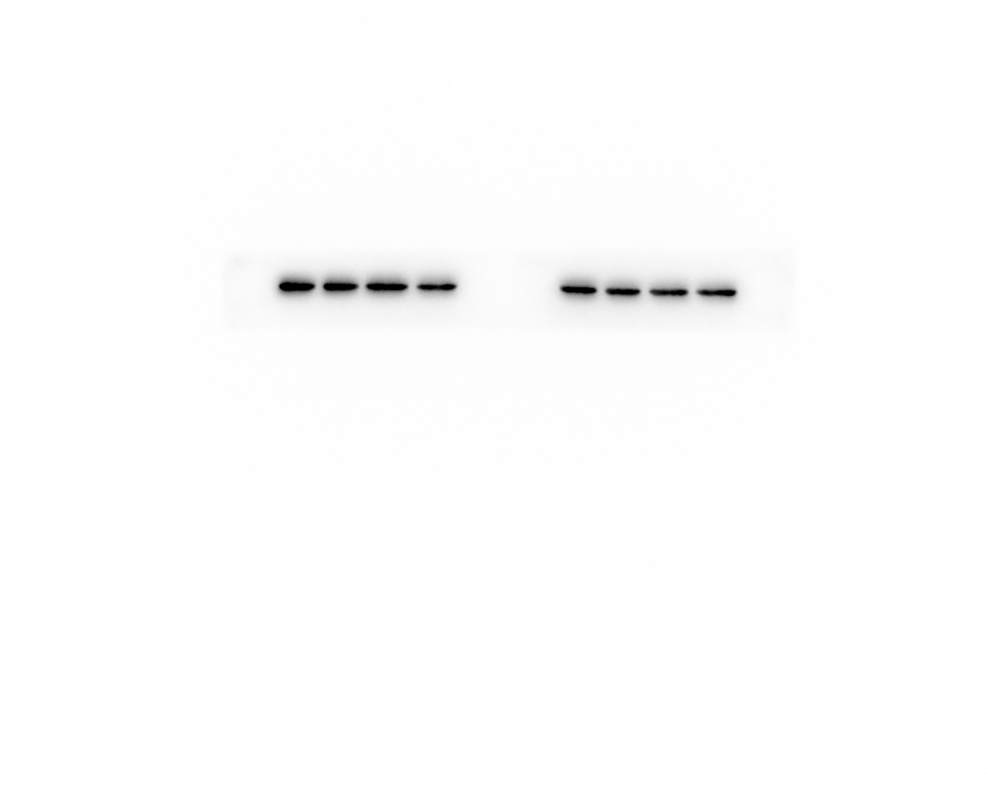


A549 cell line replication 1

p62


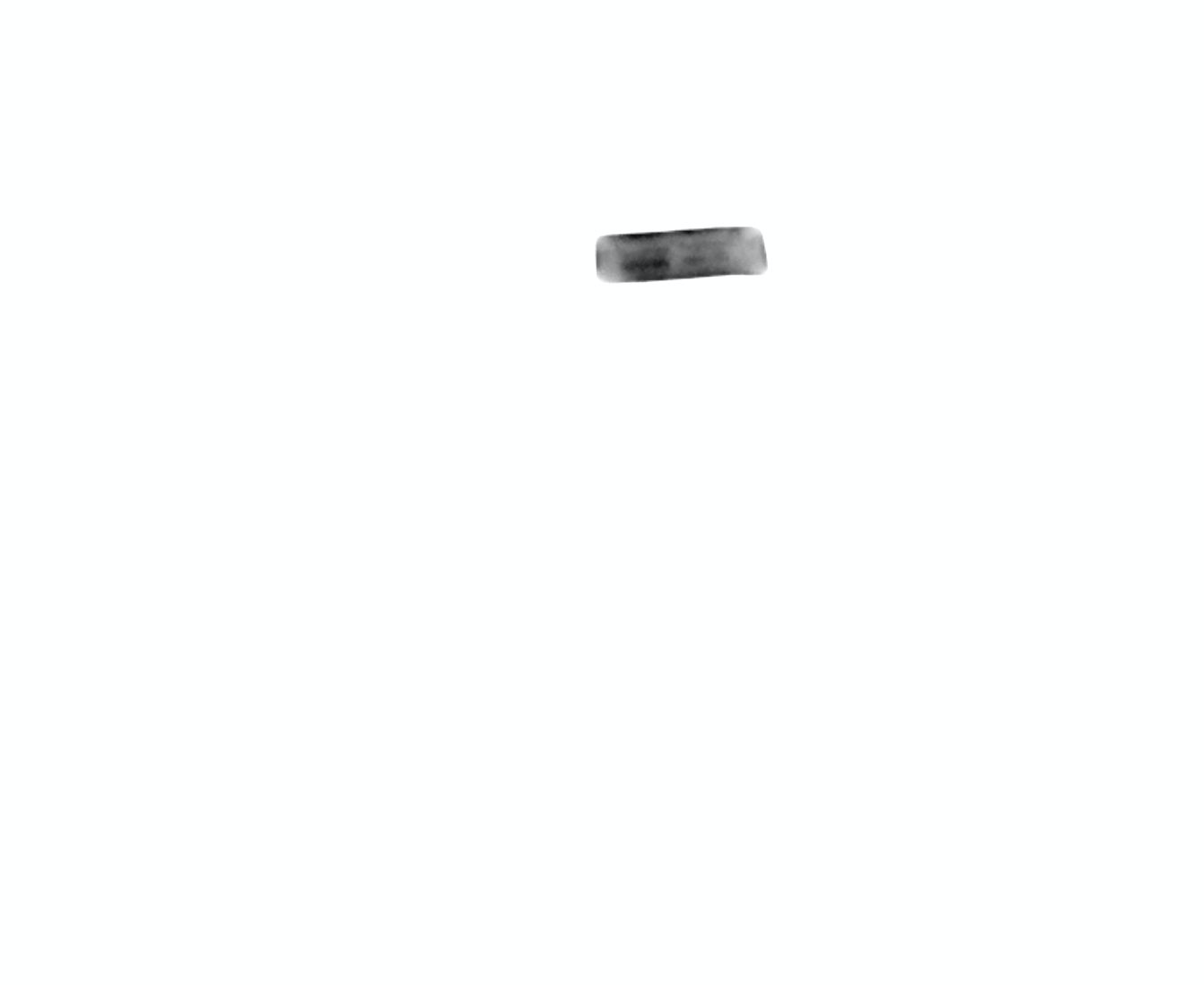


LC3B


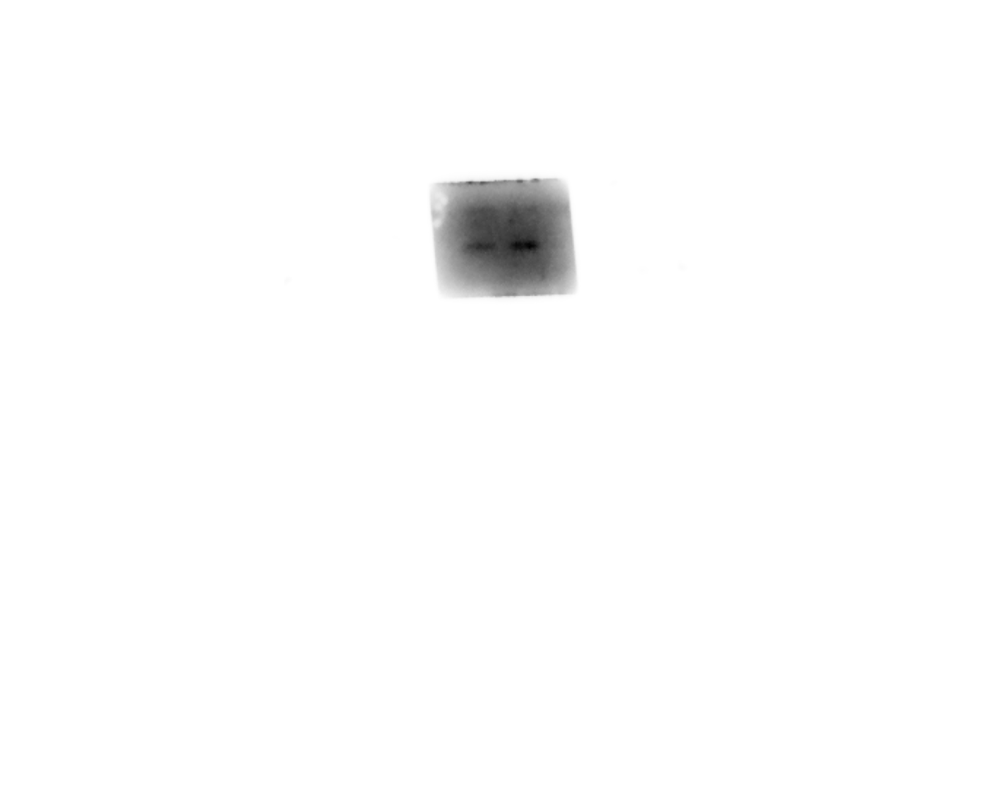

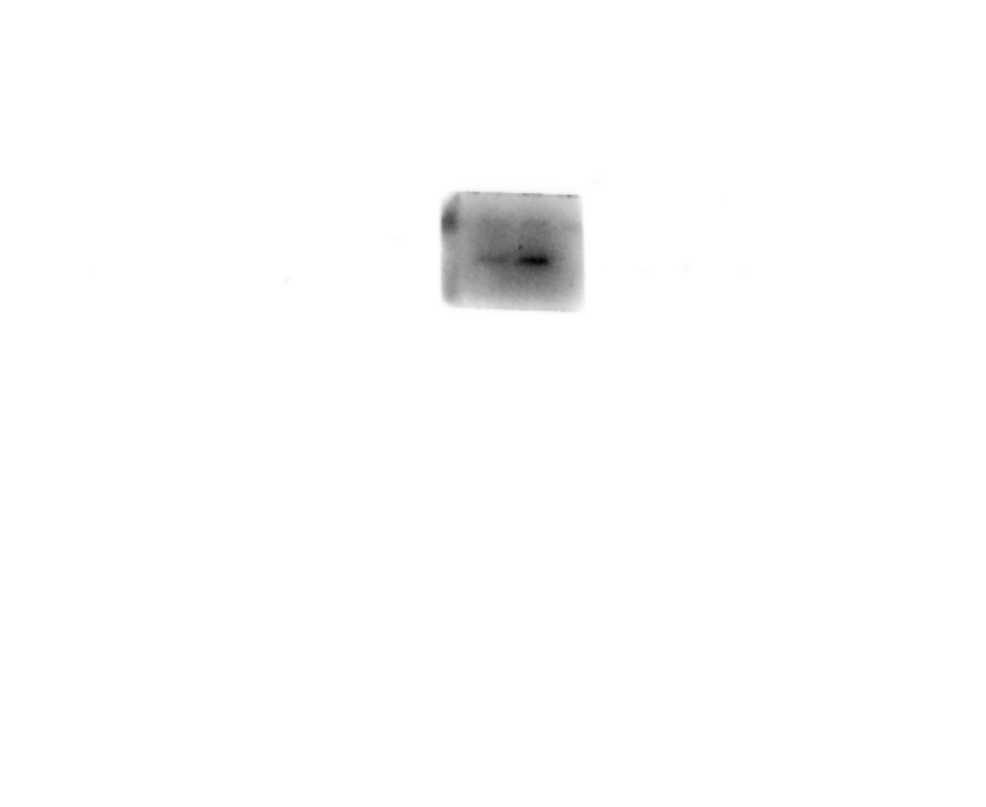


GAPDH


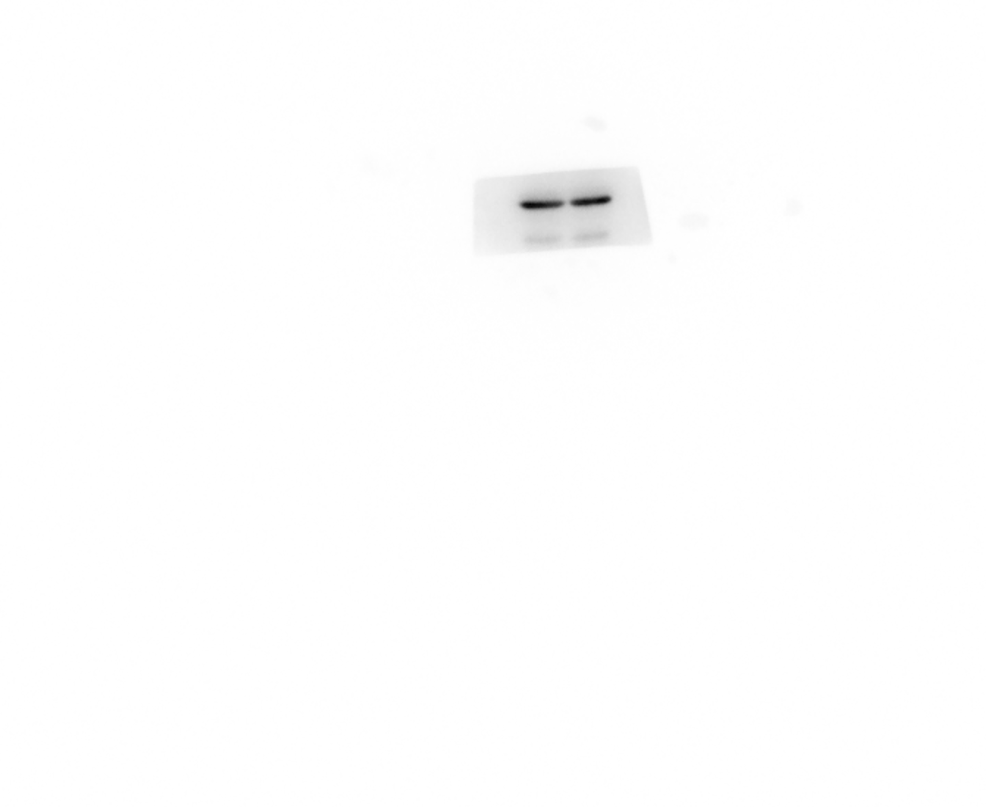


A549 cell line replication 2

p62


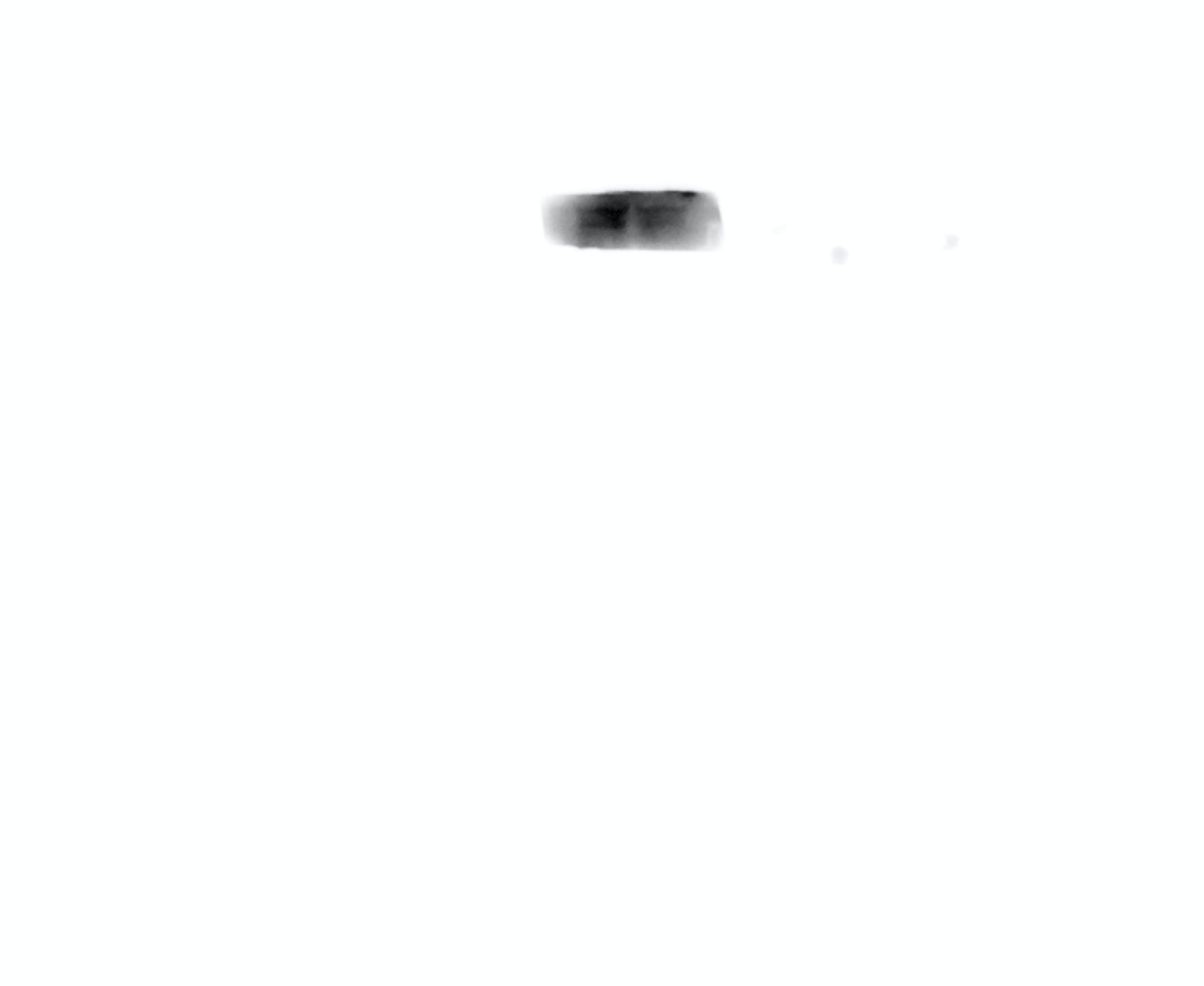


LC3B


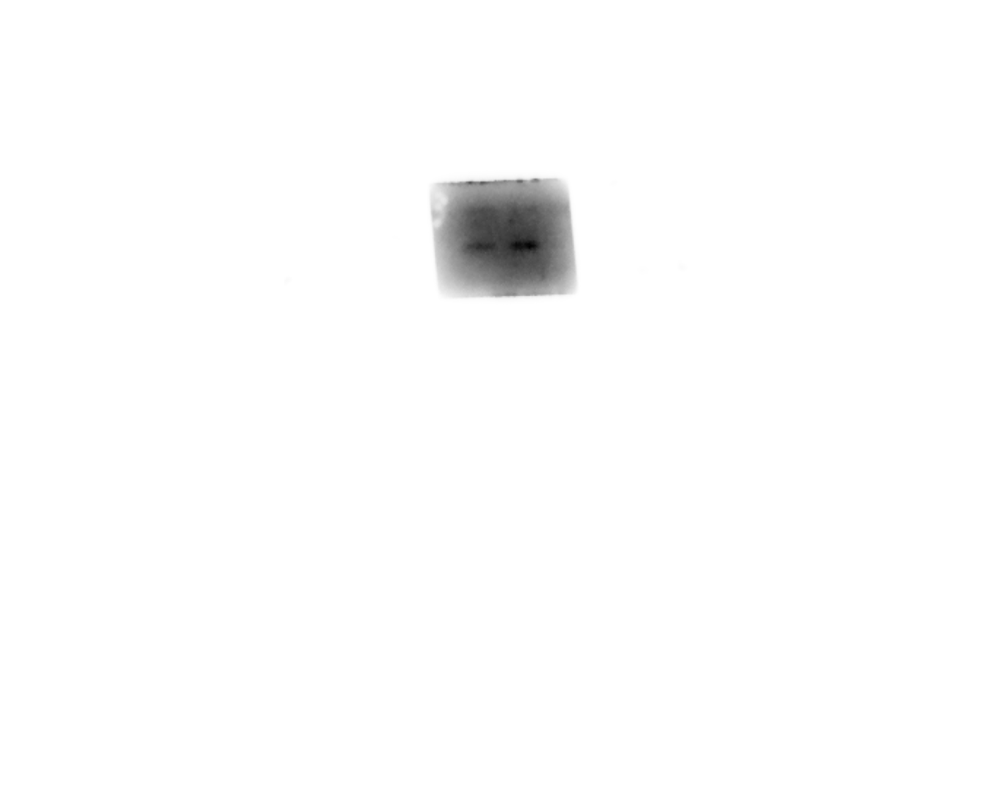


GAPDH


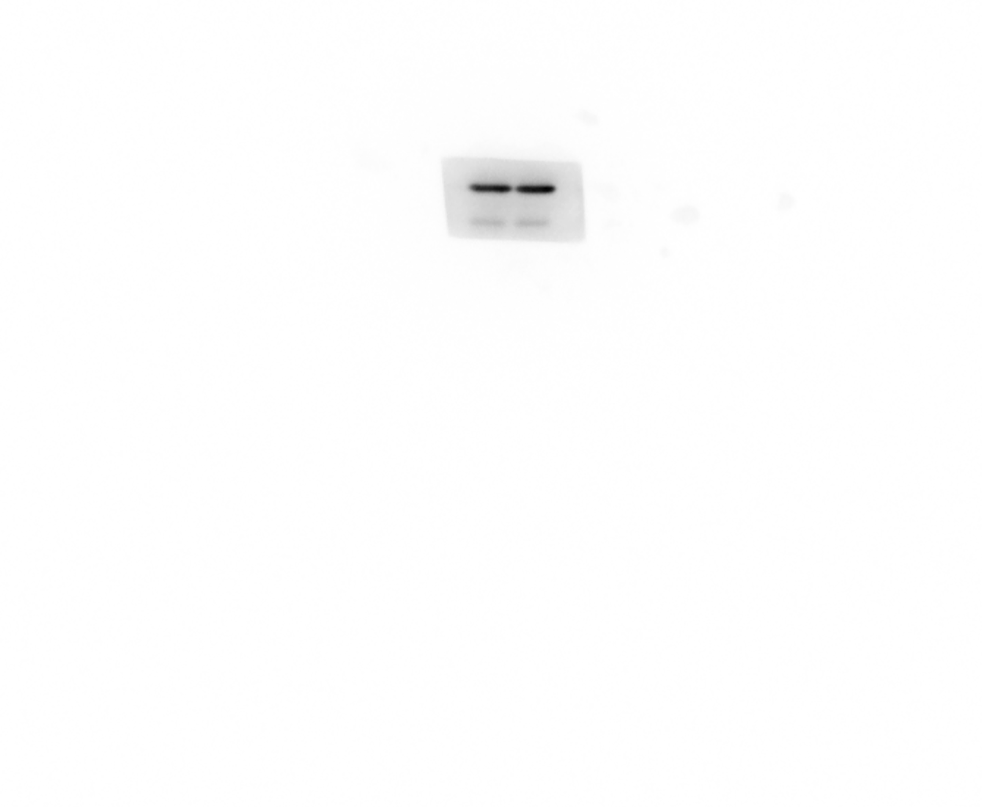


**H1975** **original blots:**

p62


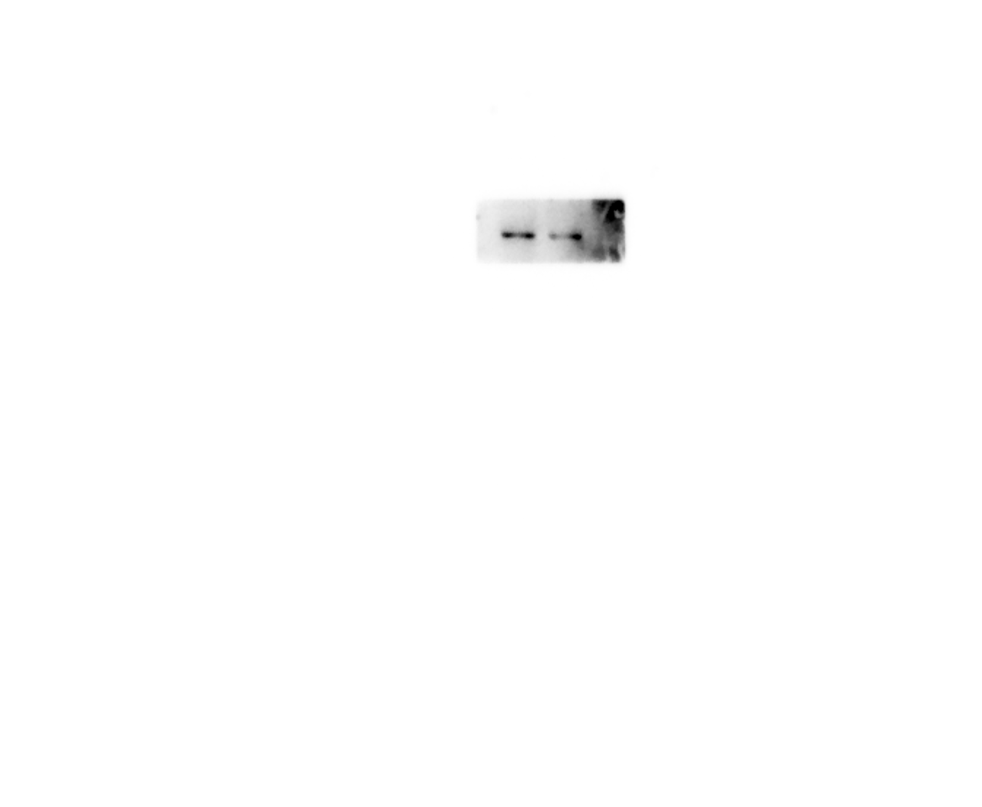


LC3B


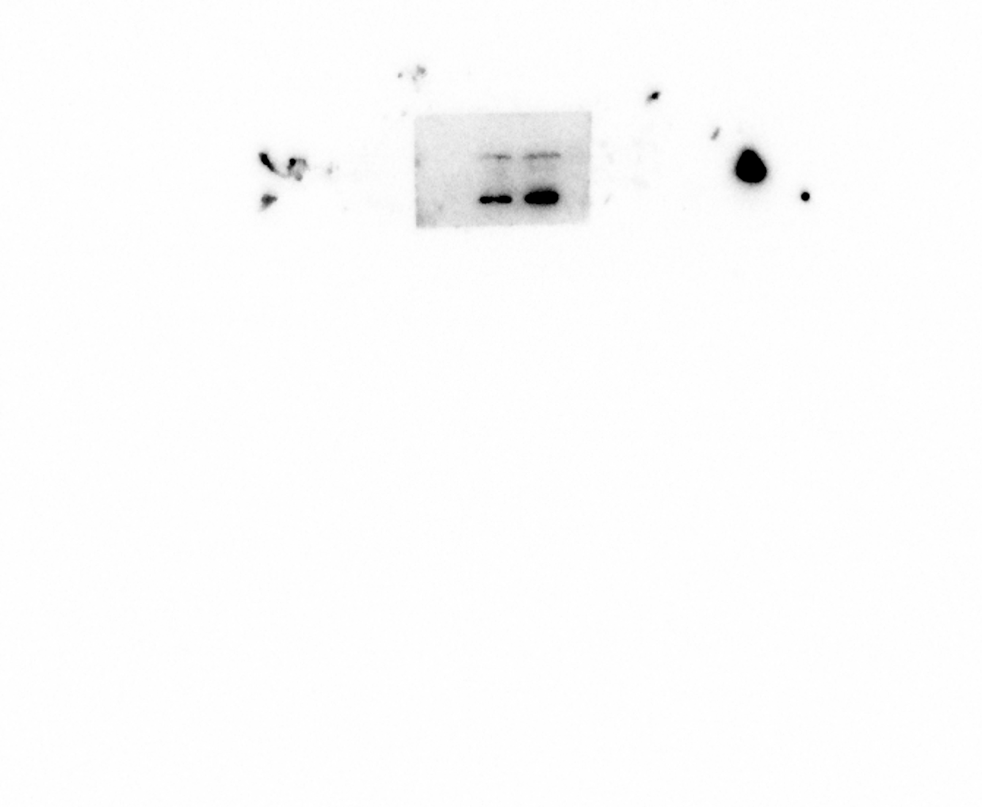


GAPDH


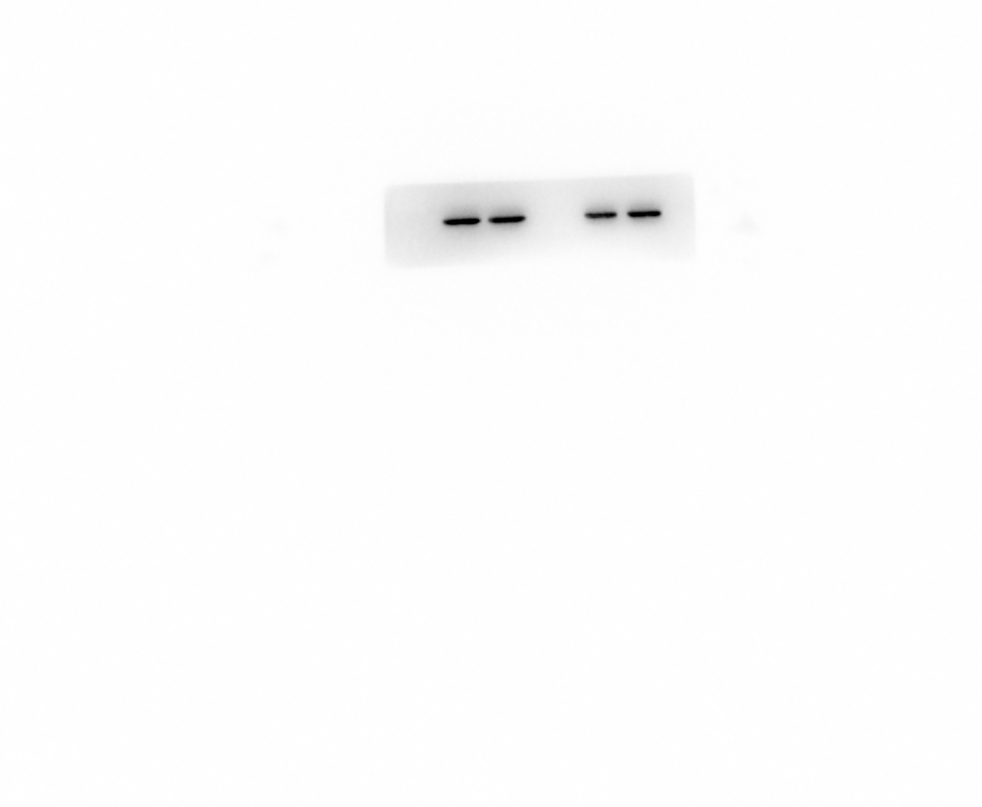


H1975 cell line replication 1

p62


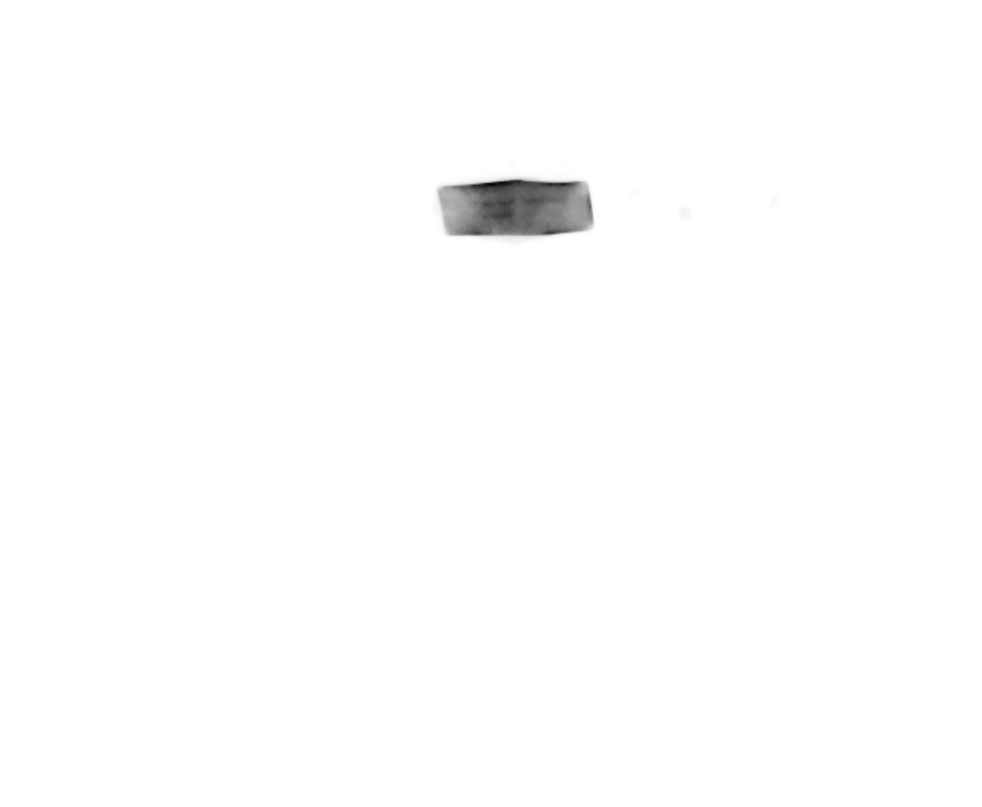


LC3B


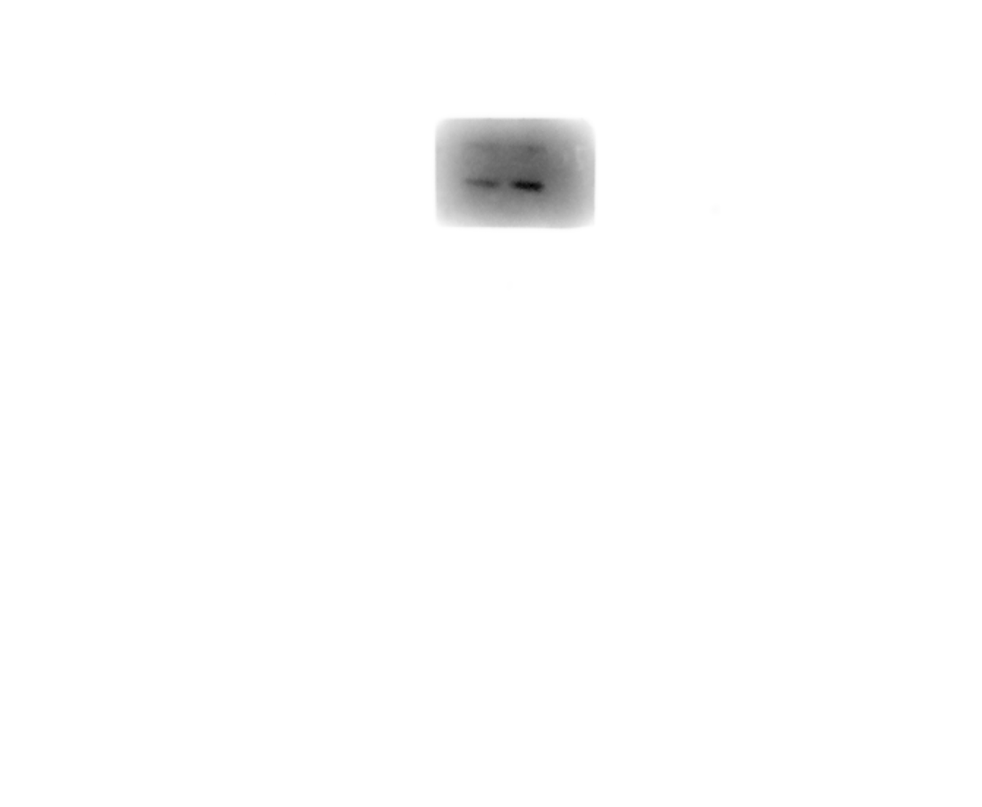


GAPDH


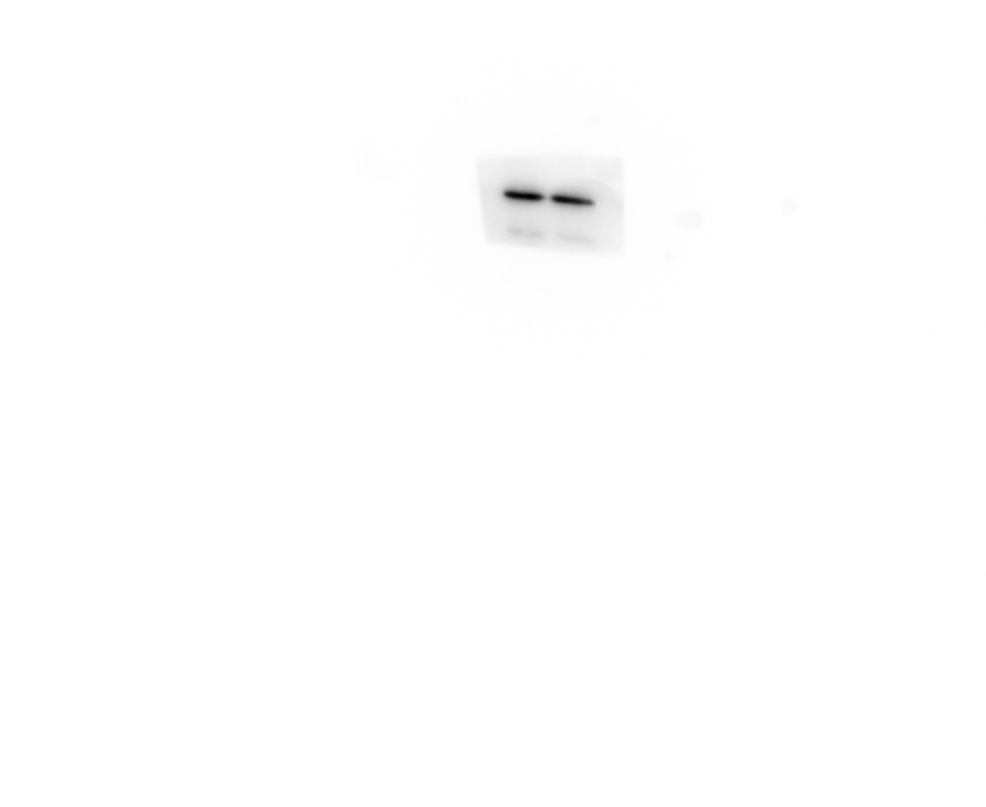


H1975 cell line replication 2

p62


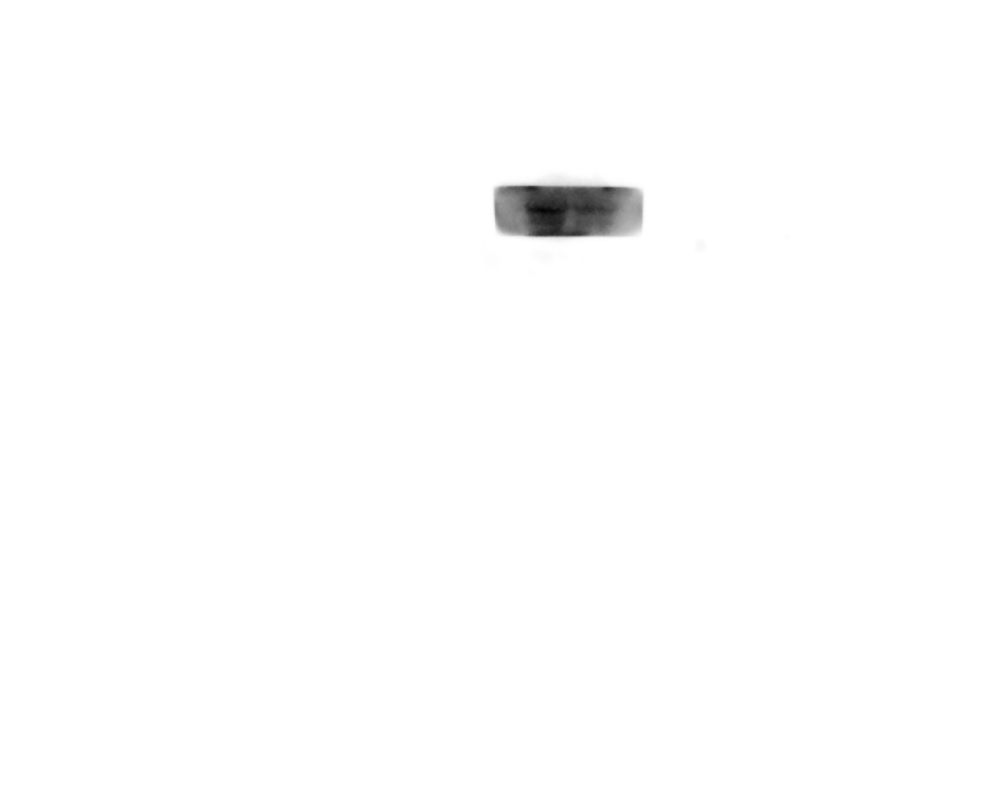


LC3B


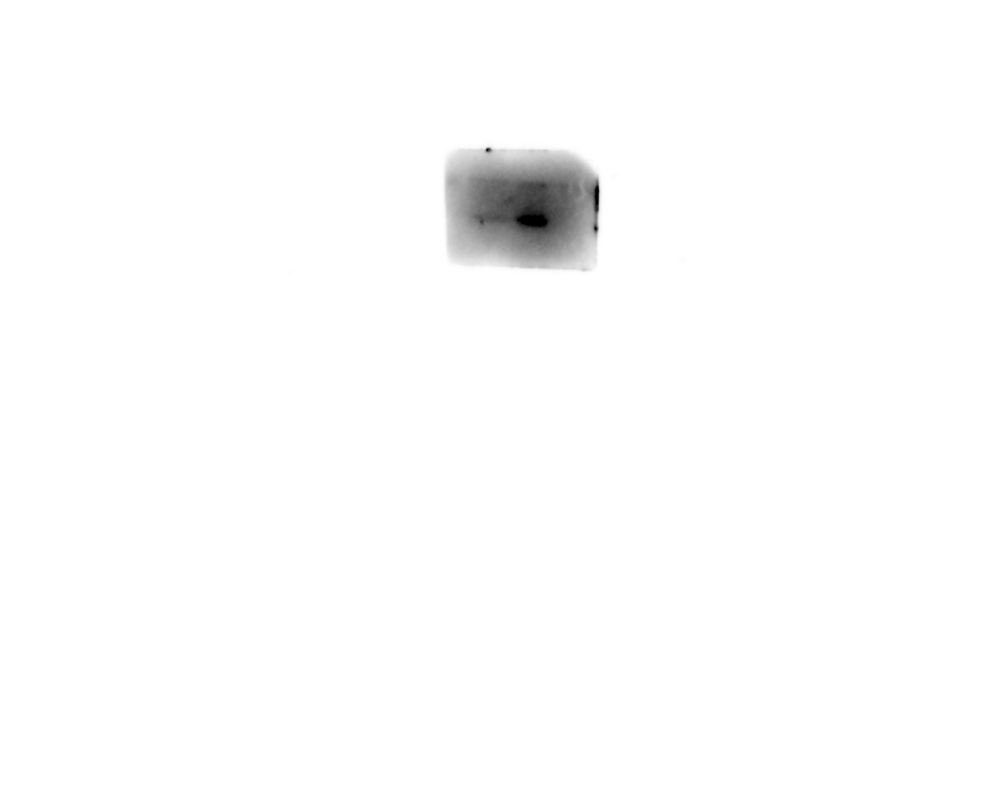


GAPDH


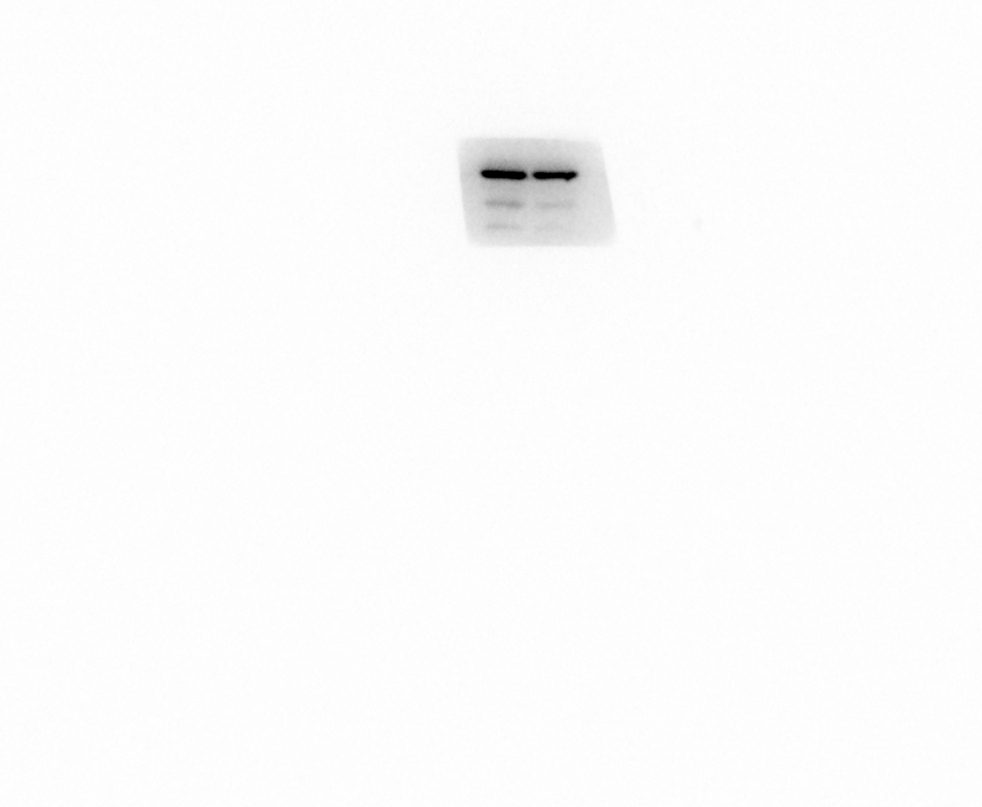

Supplement: Supplementary file 1 [file LSA-2023-02408_SdataF3.docx]
