# Supplementary material for: Linc00673-V3 positively regulates autophagy by promoting Smad3-mediated LC3B transcription in NSCLC
Source: Life Sci Alliance. 2024 Mar 25;7(6):e202302408. doi: 10.26508/lsa.202302408 (PMC10963591; doi:10.26508/lsa.202302408)
Supplement: Supplementary file 3 [file LSA-2023-02408_TableS2.docx]

**Supplementary table2. Primers for CLIP assay**

| Primers for CLIP assay | |
| --- | --- |
| Primer-F1 | ACGCGCCACGCCGCGACCATA |
| Primer-F2 | GAGACAATCCGTCCGCGGGT |
| Primer-F3 | TCTCCGAAAGGGCCGCCCCTAG |
| Primer-F4 | CGCCCTACGCGAGCGAACCA |
| Primer-F5 | GCTGGAGGCGCTCAGACCT |
| Primer-F6 | ACCTAATGAACCACACATAGTCGACC |
| Primer-F7 | TCCATAAAAAGGGAGAGGTGGGAC |
| Primer-F8 | ACCTTAGTCTCCAAAGACCGACT |
| Primer-F9 | TCGTCTTGGGGGCGCACGG |
| Primer-F10 | ACGACTCGTACCACGGAGTACG |
| Primer-F11 | TCTCTCTGGGGTAGAGATTTTAT |
| Primer-F12 | TTCTCCTACCGACCTCGGACCC |
| Primer-F13 | TTATTATTATTATTATTAGAATAAAAC |
| Primer-F14 | ACACAACGGGACTTCCTCGTCT |
| Primer-F15 | CAAAGGAGACGACGATGACGAGTAC |
| Primer-F16 | AGACGACACGGAAACATGAGTCG |
| Primer-F17 | TAAAAACAAGGACGAGGCACA |
| Primer-F18 | AGGTCTTACTATGTGCCTAGTC |
| Primer-F19 | CAAGAGGTAGGAGTTGAAAGAAACG |
| Primer-F20 | TGTCTACGTGTCAGACGACACTTC |
| Primer-F21 | CACCGTGAATCACTCCTGAGAGG |
| Primer-F22 | TGTATGTGTACTAAATTTTGGGAAA |
| Primer-F23 | GCTTATCAACCGAAAGAAAAACG |
| Primer-R | Universal PCR Primer R |

Universal PCR reserve primers for products of poly(A) tailing reaction were provided by Sangon Biotech.
