# Supplementary material for: Linc00673-V3 positively regulates autophagy by promoting Smad3-mediated LC3B transcription in NSCLC
Source: Life Sci Alliance. 2024 Mar 25;7(6):e202302408. doi: 10.26508/lsa.202302408 (PMC10963591; doi:10.26508/lsa.202302408)
Supplement: Supplementary file 4 [file LSA-2023-02408_TableS3.docx]

**Supplementary table 3. siRNA sequences**

| siRNA sequences | |
| --- | --- |
| si-Linc00673-#3-sense | 5’-GGAGUCCAUGCCAGAUCAUTT-3’ |
| si-Linc00673-#3-antisense | 5’-AUGAUCUGGCAUGGACUCCTT-3’ |
| si-Linc00673-#5-sense | 5’-GGAUACAGAGUGAAUAGUUTT-3’ |
| si-Linc00673-#5-antisense | 5’-AACUAUUCACUCUGUAUCCTT-3’ |
| si-Smad3-sense | 5’-CCCAGCACAUAAUAACUUGGA-3’ |
| si-Smad3-antisense | 5’-TCCAAGTTATTATGTGCTGGG-3’ |
| si-Nedd4L-sense | 5’-AACCACAACACAAAGUCACAG-3’ |
| si-Nedd4L-antisense | 5’-CTGTGCTTTGTGTTGTGGTT-3’ |
| si-STUB1-sense | 5’-CCCAAGTTCTGCTGTTGGACTCT-3’ |
| si-STUB1-antisense | 5’-AGAGTCCAACAGCAGAACTTGGG-3’ |
| si-VHL-sense | 5’-GGAGCGCAUUGCACAUCAATT-3’ |
| si-VHL-antisense | 5’-UUGAUGUGCAAUGCGCUCCTG-3’ |
| ASO-Linc00673 | 5’-AGAAACCTCTGATTCCACC-3’ |
