# Supplementary material for: Linc00673-V3 positively regulates autophagy by promoting Smad3-mediated LC3B transcription in NSCLC
Source: Life Sci Alliance. 2024 Mar 25;7(6):e202302408. doi: 10.26508/lsa.202302408 (PMC10963591; doi:10.26508/lsa.202302408)
Supplement: Supplementary file 5 [file LSA-2023-02408_TableS4.docx]

**Supplementary Table4. The detailed antibody information**

| **Antibodies** | **Source** | **Cat#** |
| --- | --- | --- |
| Anti-GAPDH | Proteintech | 60004-1-Ig |
| Anti-LC3B | Cell Signaling Technology | #83506 |
| Anti-p62 | Cell Signaling Technology | #23214 |
| Anti-Smad3 | Abcam | ab40854 |
| Anti-p-Smad3 | Cell Signaling Technology | #9520 |
| Anti-Ubiquitin | Cell Signaling Technology | #3936 |
| Anti-HA | Cell Signaling Technology | #3724 |
| Anti-ULK1 | Cell Signaling Technology | #8054 |
| Anti-Beclin1 | Cell Signaling Technology | #3495 |
| Anti-ATG3 | Cell Signaling Technology | #3415 |
| Anti-ATG4 | Cell Signaling Technology | #5299 |
| Anti-ATG5 | Cell Signaling Technology | #12994 |
| Anti-ATG7 | Cell Signaling Technology | #8558 |
| Anti-ATG12 | Cell Signaling Technology | #4180 |
| Anti-Nedd4L | Abclonal | A9078 |
| Anti-STUB1 | Abclonal | A11751 |
| Anti-VHL | Abclonal | A23239 |
