# Supplementary material for: Linc00673-V3 positively regulates autophagy by promoting Smad3-mediated LC3B transcription in NSCLC
Source: Life Sci Alliance. 2024 Mar 25;7(6):e202302408. doi: 10.26508/lsa.202302408 (PMC10963591; doi:10.26508/lsa.202302408)
Supplement: Supplementary file 6 [file LSA-2023-02408_TableS5.docx]

**Supplementary table5. Primers for qRT-PCR**

| Primers for qRT-PCR | |
| --- | --- |
| Linc00673-F | CCGTGTAAAGAGGCCAGTGT |
| Linc00673-R | ACACGAGCCTTCACCATCAG |
| Linc00673-V1-F | ATTTTTCCCTCTCCACCCTGG |
| Linc00673-V1-R | CCAGCCCTGTAGAGGTCCTCA |
| Linc00673-V2-F | CAAGCTGGAGGTGGAATCAGAGG |
| Linc00673-V2-R | AGGAGGTGGCACCTCTTTCTTG |
| Linc00673-V3-F | ATTTTTCCCTCTCCACCCTGG |
| Linc00673-V3-R | GTCCTTCCCATCCTCTTTCTTG |
| Linc00673-V4-F | CAAGCTGGAGGTGGAATCAGAGG |
| Linc00673-V4-R | GTCCTTCCCATCCTCTTTCTTG |
| Linc00673-V5-F | AGATCAGGTTCAAGTTGCCAG |
| Linc00673-V5-R | CTGCAGGGCCGGGAGGAGAGG |
| ATG3-F | TCAGTTCACCCATGCAGGC |
| ATG3-R | GCCACTAATCTTACATACAGGGA |
| ATG4-F | ACGTCCTGAACCTGTCCCTA |
| ATG4-R | TGGCAGTGTCGAGAGAACAC |
| ATG5-F | TCCCTCTTGGGGTACATGTCT |
| ATG5-R | CGTCCAAACCACACATCTCG |
| ATG7-F | GAGCCTCCAACCTCTCTTGG |
| ATG7-R | TCTCAGCAGCTTGGGTTTCT |
| ATG8-F | AAGGCTTTCAGAGAGACCCTG |
| ATG8-R | TCTCACACAGCCCGTTTACC |
| ATG12-F | TGCTGGAGGGGAAGGACTTA |
| ATG12-R | CACGCCTGAGACTTGCAGTA |
| Beclin1-F | GGGCTCCCGAGGGATGG |
| Beclin1-R | AGTTCCTGGATGGTGACACG |
| ULK1-F | GCGGCTCTTTTGTTTCTCCG |
| ULK1-R | CTCTTCCCGGGCTGCTAATC |
| Smad3-F | AGGGCTTTGAGGCTGTCTACC |
| Smad3-R | GTGCTGGTCACTGTCTGTCTCCT |
| Nedd4L-F | AGAAACTGCCCAGAGCTCAC |
| Nedd4L-R | TCGCCTCTGCAAAAGTCTGT |
| STUB1-F | GCAGGGCAATCGTCTGTTCG |
| STUB1-R | TTCCGGGTCTAGGGACCAAGG |
| VHL-F | CATCCACAGCTACCGAGGTC |
| VHL-R | GGCAAAAATAGGCTGTCCGTC |
| GAPDH-F | CACCCACTCCTCCACCTTTG |
| GAPDH-R | CCACCACCCTGTTGCTGTAG |
